# Independent associations between arterial bicarbonate, apnea severity and hypertension in obstructive sleep apnea

**DOI:** 10.1186/s12931-017-0607-9

**Published:** 2017-06-28

**Authors:** Davoud Eskandari, Ding Zou, Ludger Grote, Hartmut Schneider, Thomas Penzel, Jan Hedner

**Affiliations:** 1 Center for Sleep and Vigilance Disorders, Department of Internal Medicine and Clinical Nutrition, Sahlgrenska Academy, University of Gothenburg, Medicinaregatan 8B, Box 421, SE-40530 Gothenburg, Sweden; 2 Johns Hopkins Sleep Disorders Center, Division of Pulmonary and Critical Care Medicine, Johns Hopkins University, Baltimore, Maryland USA; 3 Interdisciplinary Center of Sleep Medicine, Charité-Universitätsmedizin Berlin, Berlin, Germany; 4 International Clinical Research Center, St. Anneʼs University Hospital Brno, Brno, Czech Republic

**Keywords:** Acid base, Blood pressure, Carbonic anhydrase, Hypercapnia, Obstructive sleep apnea

## Abstract

**Background:**

Obstructive sleep apnea is characterized by intermittent hypoxia and hypercapnia. CO_2_ production, transport and elimination are influenced by the carbonic anhydrase enzyme. We hypothesized that elevated standard bicarbonate, a proxy for increased carbonic anhydrase activity, is associated with apnea severity and higher blood pressure in patients with obstructive sleep apnea.

**Methods:**

A retrospective analysis of a sleep apnea cohort (*n* = 830) studied by ambulatory polygraphy. Office systolic/diastolic blood pressure, lung function, and arterial blood gases were assessed during daytime.

**Results:**

Arterial standard bicarbonate was increased with apnea severity (mild/moderate/severe 24.1 ± 1.8, 24.4 ± 1.7 and 24.9 ± 2.9 mmol/l, respectively, Kruskal-Wallis test *p* < 0.001). Standard bicarbonate was independently associated with apnea hypopnea index after adjustment for sex, age, body mass index, smoking, alcohol, hypertension, pO_2_ and pCO_2_ (standard bicarbonate quartile 1 vs. quartile 4, β = 10.6, *p* < 0.001). Log-transformed standard bicarbonate was associated with a diagnosis of hypertension or diastolic blood pressure but not systolic blood pressure adjusting for cofounders (*p* = 0.007, 0.048 and 0.45, respectively).

**Conclusions:**

There was an independent association between sleep apnea severity and arterial standard bicarbonate. The link between high standard bicarbonate and daytime hypertension suggests that carbonic anhydrase activity may constitute a novel mechanism for blood pressure regulation in sleep apnea.

**Electronic supplementary material:**

The online version of this article (doi:10.1186/s12931-017-0607-9) contains supplementary material, which is available to authorized users.

## Background

Obstructive sleep apnea (OSA) is associated with intermittent oscillations of oxygen and carbon dioxide (CO_2_) during the sleeping period. The severity of these changes is determined not only by altered ventilation during the apneic cycle, but also by the extent of tissue oxidative metabolism and tissue deposition of CO_2_ in the body [1]. CO_2_ production, transport and elimination are influenced by the activity of the enzyme carbonic anhydrase (CA). CA catalyzes the inter-conversion of CO_2_ and water into carbonic acid, protons and bicarbonate (StHCO_3_^-^) [2]. Hence, this enzyme plays an important role for the maintenance of blood gas stability in OSA. We have previously demonstrated an association between whole blood CA activity and the severity of OSA [3]. Further we hypothesized that arterial StHCO_3_^-^ concentration (a surrogate for CA activity) was elevated in relation to the degree of disordered breathing in OSA patients.

Hypertension development in OSA has been related to multiple mechanisms including increased sympathetic autonomic activity, endothelial dysfunction and modified activity of the renin-angiotensin aldosterone system [4]. Hypoxic and/or hypercapnic chemoreceptor reflex activation and modified baroreflex sensitivity have also been implied in OSA related hypertension [5]. In fact, hypercapnia may be of greater importance than hypoxia in causing sympathetically induced blood pressure elevation and vascular resistance in OSA [6]. In addition, results from in-vitro and in-vivo protocols suggest an association between CA activity and blood pressure controlling mechanisms [7, 8]. Hypertensive OSA patients reduced blood pressure in response to pharmacological inhibition of CA [9] and acetazolamide partially prevented blood pressure elevation in OSA patients moving from low- to high altitude [10]. Whether the surrogate for CA activity, StHCO_3_^-^, is associated with hypertension in OSA patients independent of sleep apnea severity has never been investigated. In the current study, we aimed to address the association between StHCO_3_^-^ and OSA activity as well as to examine a possible link between StHCO_3_^-^ and hypertension.

## Methods

### Study population and protocol

The study cohort (*n* = 1656) consisted patients successively referred to the Marburg Sleep Disorders Center between 1989–1992 due to clinical symptoms of sleep related breathing disorder [11]. Patients were systematically investigated to study the relationship between OSA, lung function and hypertension. In detail, anthropometric data such as age, sex, body mass index (BMI) were collected. Alcohol consumption, smoking habits along with signs of sleep disorder, such as excessive daytime sleepiness, snoring and insomnia were also recorded. In addition, a medical history including clinical diagnoses and concomitant medication, with particular focus on known and/or treated hypertension, was obtained. Daytime arterial blood samples for determination of blood gases, including StHCO_3_^-^, were collected. A home sleep study and lung function test using full body plethysmography (Jäger, Würzburg, Germany) were undertaken. All patients gave their written consent for participation in the study according to the contemporary regulations for medical research at the Marburg University, Marburg, Germany in 1989–1992.

### Exclusion criteria for data analysis

**Fig. 1:**
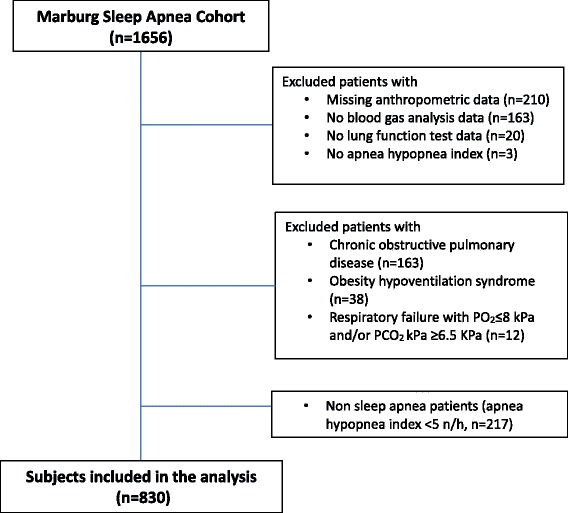
Study flow chart

Three hundred ninety six patients were excluded from the analysis due to missing anthropometric data (*n* = 210), blood gas data (*n* = 163), lung function test data (*n* = 20) and sleep data (*n* = 3). Patients with obesity hypoventilation syndrome (*n* = 38, defined as BMI > 30 kg/m^2^ with a pCO_2_ > 6 kPa), chronic obstructive pulmonary disease, defined as the ratio of forced expiratory volume in 1 s to forced vital capacity of less than 0.70, were excluded (*n* = 163). In addition, 12 respiratory failure patients with arterial blood gas pO_2_ ≤ 8.0 kPa and/or pCO_2_ ≥ 6.5 kPa were excluded. Finally, non-OSA patients with apnea hypopnea index (AHI) <5 n/h were excluded from the analysis (*n* = 217) and a total of 830 patients were included in the data analysis (Fig. 1).

### Blood pressure, blood gas and blood sample assessment

Systolic (SBP) and diastolic (DBP) blood pressures were obtained with the patient in a sitting position after a minimum of 10 minutes rest, between 9.00 and 11.00 a.m., using the World Health Organization standard protocol [12]. Heart rate defined as beats per minute was determined in the sitting position by pulse wave palpation. Blood samples were obtained at the morning following an overnight fast. A blood gas analysis (samples obtained at noon) was performed using a RADIOMETER®- gas analyzer (Radiometer, Copenhagen, Denmark).

### Definition of hypertension

Hypertension was defined as patients with a previous positive history of diagnosed hypertension and/or on ongoing hypertensive medication. Normotension was defined as patients with no previous hypertensive medical history and no ongoing anti-hypertensive treatment.

### Sleep study

All patients underwent unattended home monitoring of nocturnal breathing on two consecutive nights using the MESAM 4 polygraphy device (MAP®, Munich, Germany). The polygraphy system was applied in the afternoon for a recording span between 6.00 p.m. and 8.00 a.m. Time to bed, lights out, final awakening, longer periods of sleep interruption and estimated sleep time were assessed using a patient diary. The first night was an adaptation night and the AHI value used for calculation in this study was obtained from the second night. Only when the recording was technically insufficient (<10% of recordings) or the subjective sleep time was <5 hours (<1% of recordings), then AHI was obtained from the first night.

The MESAM 4 device records oxygen saturation using finger pulse oximetry, snoring using an electret-miniature microphone placed over the larynx, beat to beat heart rate analysis using ECG, and body position using a circular sensor taped just below the sternum. It is a validated sleep diagnostic instrument for clinical and epidemiological studies [13, 14]. In the current study, apnea hypopnea events were determined visually using the previously described methods of MESAM 4 evaluation [15]. In detail, events were first scored with a concomitant oxygen desaturation of ≥ 4% from baseline. Subsequently, the scorers edited this information by checking the heart rate (significant drop and reduced variability when going to bed, abrupt increase and plateau after final awakening), movement artefacts, and body position signal (e.g. change from upright to supine position). Estimated sleep time was determined based on the information from the sleep diary (time going to bed, lights out, final awakening, lights on, longer periods of sleep interruption). Finally, AHI was calculated as the number of apnea/hypopnea events per hour of edited recording time. OSA severity was defined as mild (5 ≤ AHI < 15 n/h), moderate (15 ≤ AHI < 30 n/h) and severe (AHI ≥ 30 n/h).

### Statistics

Statistical analysis was conducted using IBM SPSS 20 (SPSS Inc, Chicago, USA). Kolmogorov-Simirnov test was used to determine the distribution of the data. Spearman correlation was used to study the association between arterial StHCO_3_^-^ and AHI, SBP and DBP. Fisher exact test was used to compare catergory variables. Depending on data distribution, differences across apnea severity groups and StHCO_3_^-^ quartiles were assessed by one-way analysis of variance (ANOVA) or Kruskal-Wallis test. Multivariate generalized linear models were used to address the independent association between StHCO_3_^-^, apnea severity and hypertension. StHCO_3_^-^ was log-transformed in order to enable parametric statistics. Data are presented as mean ± SD. A *p*-value <0.05 was considered statistically significant.

## Results

### Study population and blood gas characteristics

In total 830 patients were included in the study (93.3% men, age 51 ± 10 years, BMI 30 ± 5 kg/m^2^, AHI 32 ± 24 n/h). Hypertension was prevalent in 53.3% of the patients and increased with OSA severity class. Patient characteristics in different OSA severity classes are shown in Table 1. There was a small but significant change in arterial blood gases in higher AHI severity classes. Mean pO_2_ decreased with approximately 0.5 kPa from mild to severe OSA patients (*p* < 0.001). Mean pCO_2_ increased slightly in patients with severe OSA (*p* = 0.046). The pH and lung function values did not change along with severity class of OSA.

**Table 1:** Patient characteristics across sleep apnea severity

	Mild *n* = 272	Moderate *n* = 211	Severe *n* = 347	*P* value (ANOVA)
Male sex (%)	89	95	96	0.001*
Age (yrs)	50 (11)	51 (9)	52 (9)	0.10
Body mass index (kg/m^2^)	28 (4)	29 (4)	32 (6)	<0.001
Smoking (%)	28	29	28	0.98*
Systolic BP (mmHg)	141 (18)	145 (21)	152 (22)	<0.001
Diastolic BP (mmHg)	91 (12)	94 (12)	97 (13)	<0.001
Heart rate (bpm)	71 (11)	71 (10)	77 (11)	<0.001
Hypertension (%)	40.4	51.7	64.3	<0.001*
Apnea hypopnea index (n/h)	9 (3)	22 (4)	55 (18)	<0.001
FEV_1_/FVC (%)	82 (6)	81 (5)	81 (6)	0.33
pH	7.41 (0.02)	7.42 (0.02)	7.42 (0.03)	0.075
pO_2_ (kPa)	10.8 (1.0)	10.7 (1.0)	10.3 (1.0)	<0.001
pCO_2_ (kPa)	5.06 (0.40)	5.07 (0.36)	5.13 (0.39)	0.046
StHCO_3_^-^ (mmol/l)	24.1 (1.8)	24.4 (1.7)	24.9 (2.9)	<0.001^#^

*Fisher exact test; ^#^Kruskal-Wallis test; BP = blood pressure; bpm = beat per minute; FEV1/FVC = forced expiratory volume at 1 second interval/forced vital capacity; pO_2_ = arterial partial pressure of oxygen; pCO_2_ = arterial partial pressure of carbon dioxide; StHCO_3_^-^ = arterial standard bicarbonate

### The association between StHCO_3_^-^ and sleep apnea

**Fig. 2:**
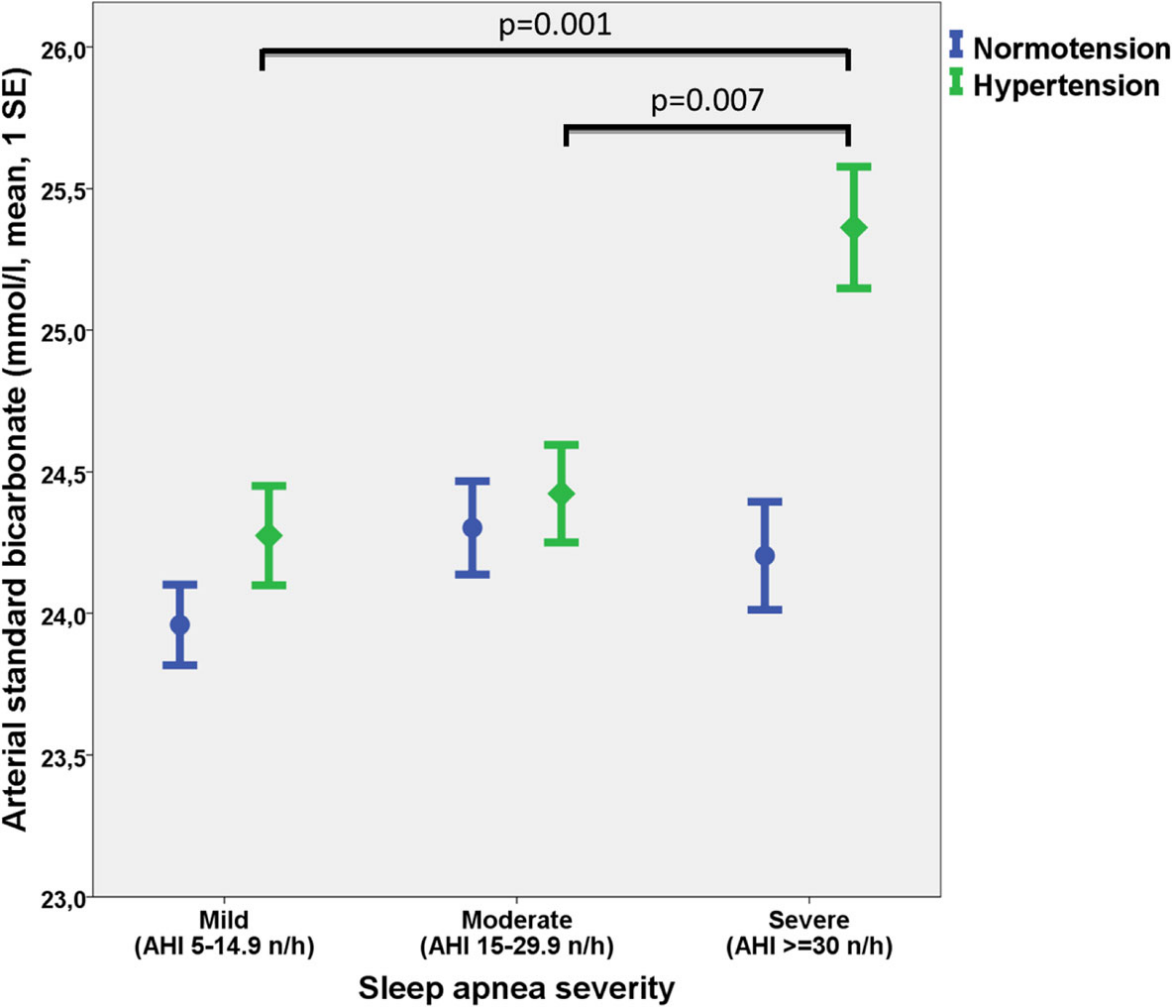
Arterial standard bicarbonate concentrations by sleep apnea severity class

Arterial StHCO_3_^-^ was significantly correlated with pCO_2_ (Spearman correlation *r* = 0.75, *p* < 0.001, Additional file 1: Figure S1). StHCO3^-^ increased across OSA severity classes although the mean absolute magnitude of change was moderate (Kruskal-Wallis test, *p* < 0.001, Table 1). The association between OSA severity and StHCO_3_^-^ was observed in hypertensive OSA patients but not in normotensive OSA patients (Fig. 2). StHCO_3_^-^ was positively correlated with AHI (Spearman correlation *r* = 0.16, *p* < 0.001, Additional file 1: Figure S2). Mean AHI increased from 27 ± 21 n/h (StHCO_3_^-^ quartile 1) to 39 ± 26 n/h (StHCO_3_^-^ quartile 4) in the whole population (*p* < 0.001, Table 2). A similar but nonsignificant trend was found in OSA patients without a hypertension diagnosis (*n* = 388, *p* = 0.094, Additional file 1: Table S1). In a generalized linear model controlling for sex, age, BMI, smoking, alcohol consumption, pO_2_, pCO_2_ and hypertension status, StHCO_3_^-^ was independently associated with AHI (Q1 vs. Q4 *β* = 10.6, *p* < 0.001, Table 3).

**Table 2:** Patient characteristics across StHCO_3_^-^ quartiles (*n* = 830)

	Q1 [17.5–23.2] mmol/l	Q2 [23.3–24.5] mmol/l	Q3 [24.6–25.7] mmol/l	Q4 [25.8–46.1] mmol/l	*P* value (ANOVA)
Male sex (%)	92	92	97	93	0.081*
Age (yrs)	51 (10)	51 (9)	49 (9)	52 (10)	0.058
Body mass index (kg/m^2^)	29 (5)	30 (5)	30 (5)	31 (5)	0.071
Smoking (%)	26	30	33	25	0.27*
Systolic BP (mmHg)	144 (19)	144 (21)	147 (22)	150 (22)	0.006
Diastolic BP (mmHg)	92 (11)	93 (13)	95 (13)	96 (13)	0.005
Heart rate (bpm)	73 (11)	72 (11)	73 (11)	75 (12)	0.065
Hypertension (%)	46.8	49.8	54.3	62.7	0.007*
Apnea hypopnea index (n/h)	27 (21)	31 (22)	30 (24)	39 (26)	<0.001
FEV_1_/FVC (%)	82 (6)	81 (6)	82 (6)	82 (6)	0.90
pH	7.41 (0.03)	7.41 (0.02)	7.42 (0.02)	7.43 (0.02)	<0.001
pO_2_ (kPa)	10.9 (1.1)	10.6 (0.9)	10.6 (1.0)	10.2 (1.0)	<0.001
pCO_2_ (kPa)	4.67 (0.30)	5.03 (0.24)	5.26 (0.24)	5.43 (0.25)	<0.001
StHCO_3_^-^ (mmol/l)	22.0 (1.1)	23.9 (0.4)	25.2 (0.3)	27.2 (2.6)	-

*Fisher exact test; *BP* blood pressure, *bpm* beat per minute, *FEV1/FVC* forced expiratory volume at 1 second interval/forced vital capacity, *pO*_*2*_ arterial partial pressure of oxygen, *pCO*_*2*_ arterial partial pressure of carbon dioxide, *StHCO*_*3*_^*-*^ arterial standard bicarbonate

**Table 3:** Generalized linear model investigating the association between apnea hypopnea index and arterial bicarbonate quartiles controlled for confounding factors

	Beta value	Standard error	95% confidence interval	*P*-value
Male sex	8.28	3.03	2.35 – 14.21	0.006
Age (years)	0.12	0.08	–0.05 – 0.28	n.s.
Body mass index (kg/m^2^)	1.45	0.16	1.14 – 1.76	<0.001
Smoking	4.37	1.71	1.03 – 7.72	0.01
Alcohol	2.00	1.58	–1.11 – 5.10	n.s.
Hypertension	4.89	1.54	1.86 – 7.92	0.002
PO_2_ (kPa)	–0.36	0.10	–0.57 – -0.16	0.001
PCO_2_ (kPa)	–0.52	0.38	–1.27 – 0.23	n.s.
StHCO_3_^-^ Q2 vs. Q1	4.76	2.29	0.27 – 9.25	0.038
StHCO_3_^-^ Q3 vs. Q1	4.34	2.67	–0.90 – 9.57	n.s.
StHCO_3_^-^ Q4 vs. Q1	10.63	3.00	4.76 – 16.51	<0.001

### Association between StHCO_3_^-^, hypertension and office blood pressure

**Fig. 3:**
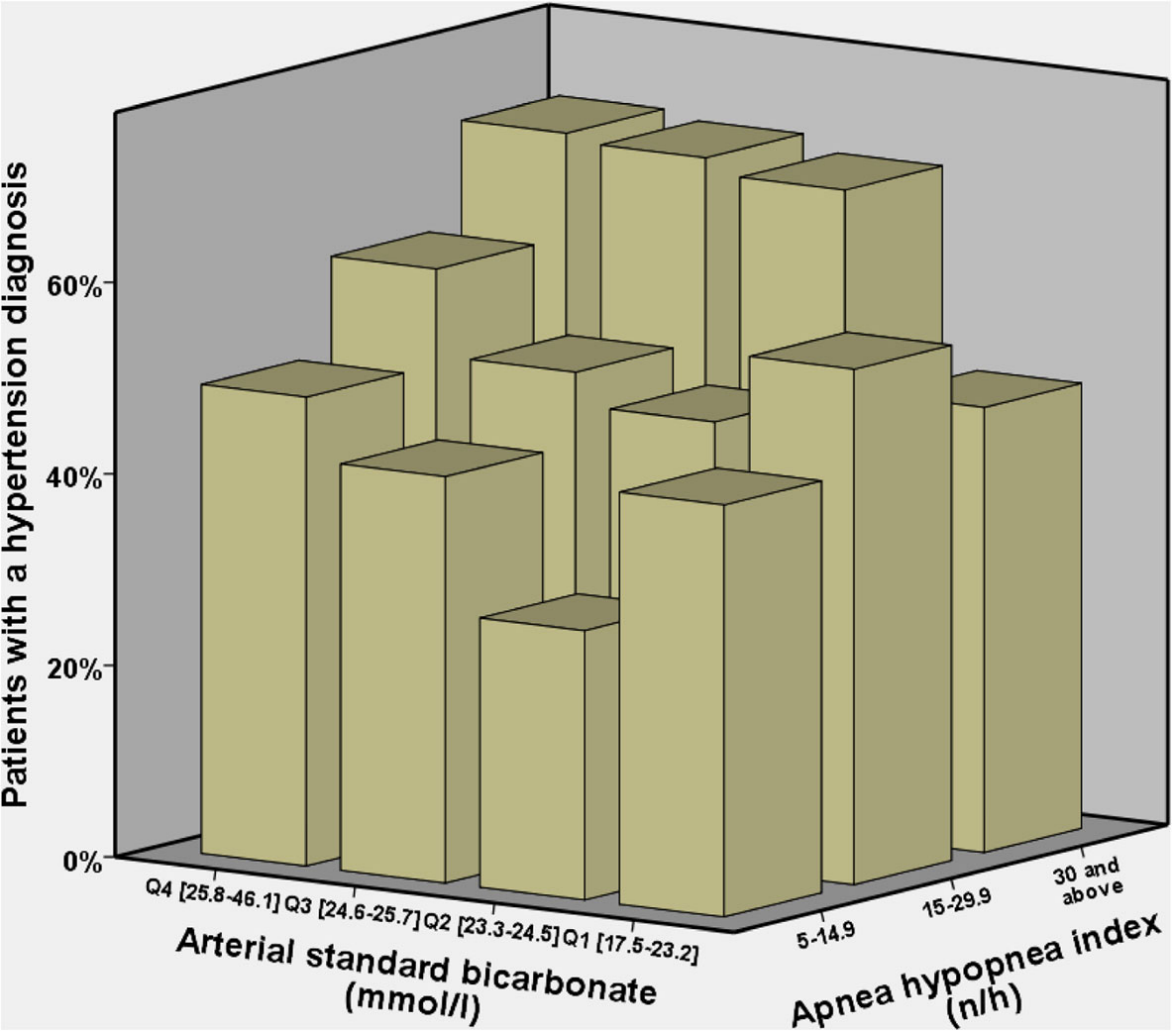
Prevalence of hypertension by sleep apnea severity and arterial standard bicarbonate quartile

StHCO_3_^-^ was higher in hypertensive (*n* = 442) compared with normotensive (*n* = 388) patients (24.9 ± 2.7 vs. 24.1 ± 1.9 mmol/l, *p* < 0.001). The percentage of patients with a clinical hypertension diagnosis is 46.8, 49.8, 54.3 and 62.7% respectively across StHCO_3_^-^ quartiles (Q1 to Q4) (*p* = 0.007, Table 2). Both OSA severity and higher StHCO_3_^-^ were associated with increased prevalence of hypertension in this population (Fig. 3). In a generalized linear model controlling for sex, age, BMI, smoking, alcohol consumption, pO_2_, pCO_2_ and apnea severity, LogStHCO_3_^-^ was independently associated with a clinical diagnosis of hypertension (*β* = 8.0, SE 3.0, 95% CI [2.1–13.8], *p* = 0.007, Table 4).

**Table 4:** Generalized linear model investigating the association between hypertension status and log-transformed arterial bicarbonate controlled for confounding factors

	Beta value	Standard error	95% confidence interval	*P*-value
Male sex	0.33	0.31	–0.28 – 0.94	n.s.
Age (years)	0.019	0.009	0.002 – 0.036	0.025
Body mass index (kg/m^2^)	0.09	0.02	0.06 – 0.13	<0.001
Smoking	–0.35	0.17	–0.69 – –0.02	0.039
Alcohol	0.16	0.16	–0.15 – 0.47	n.s.
Moderate vs. mild OSA	0.33	0.19	–0.05 – 0.70	0.088
Severe vs. mild OSA	0.52	0.18	0.16 – 0.88	0.004
PO_2_ (kPa)	–0.01	0.01	–0.04 – 0.01	n.s.
PCO_2_ (kPa)	–0.02	0.04	–0.09 – 0.05	n.s.
LogStHCO_3_^-^	7.96	2.97	2.15 – 13.77	0.007

An additional analysis was performed to study the relationship between StHCO_3_^-^ and office blood pressure. StHCO_3_^-^ was modestly correlated with SBP and DBP (Spearman correlation, *r* = 0.10 and 0.12, *p* = 0.003 and <0.001, respectively). In generalized linear models with SBP and DBP, respectively, as dependent variables, a positive independent association was found between LogStHCO_3_^-^ and DBP (β = 27.6, SE 14.0, 95% CI [0.2–55.0], *p* = 0.048) but not with SBP (β = 17.1, SE 22.3, 95% CI [-26.7–60.9], *p* = 0.45) after adjustment for sex, age, BMI, smoking, alcohol usage, pO_2_, pCO_2_ and apnea severity (Additional file 1: Table S2 and S3).

## Discussion

In this large cross sectional study of a predominantly male clinical sleep apnea cohort, we established an independent association between wake arterial StHCO_3_^-^ concentration and the intensity of sleep apnea. In addition, there was an independent association between StHCO_3_^-^ and hypertension as well as daytime office DBP. Our data suggest that mechanisms related to acid-base balance may link to the expression of OSA and its cardiovascular sequels. Given the strong inter-correlation between StHCO_3_^-^ and CA activity we speculate that CA activity is involved in blood pressure regulation in OSA patients.

Elevated StHCO_3_^-^ concentration has been used as marker for hypercapnia in patients with respiratory disorders such as obesity-hypoventilation syndrome (OHS) and pre-clinical OHS [16, 17]. The association between StHCO_3_^-^ and OSA is less well studied. In this study we excluded patients with chronic obstructive pulmonary disease and OHS based on information of a gold- standard evaluation of respiratory function and a blood gas analysis. Our data unequivocally demonstrated a dose dependent association between OSA and daytime arterial StHCO_3_^-^ concentration in this group of patients without a chronic respiratory disorder. Although the magnitude of the StHCO_3_^-^ elevation across the spectrum of OSA severity may be considered as rather limited, the association was statistically significant and remained after extensive control of important confounders. To our knowledge this is the first study to demonstrate this association in a well characterized clinical OSA cohort. The exact mechanism behind this finding in OSA remains unknown. Severe OSA may lead to mild nocturnal hypercapnia. The long term effect of OSA on PCO_2_ (i.e. StHCO3^-^) is determined by the net change of CO_2_ over each cycle of apnea/hyperventilation and asymmetry in how the rise and fall of CO_2_ affects the kidney. It is likely that changes of CA activity can modulate the transition of obstructive apnea/ventilation cycle and influence the increase in StHCO3^-^ [18–20]. We therefore propose that transient hypercapnic episodes during sleep in patients with more severe OSA lead to increased renal reabsorption of StHCO_3_^-^ and/or that a chronic increase of StHCO_3_^-^ production [18] is induced by high or possibly even up-regulated CA activity.

StHCO_3_^-^ concentration is known to be influenced by CA enzyme activity. One major function of this enzyme includes the catalysis of the interconversion of bicarbonate and protons into CO_2_ and water for subsequent removal of CO_2_ via the respiratory apparatus [2]. In OSA, repetitive changes in pCO_2_ may induce CA enzyme activity and increase arterial StHCO_3_^-^ concentration. Alternatively, anaerobic metabolism and respiratory acidosis following intermittent hypoxia may induce an increased activity of enzymes and transporters involved in cellular pH regulation and erythrocyte acid-base handling [21]. In this manner both hypercapnia and hypoxia may contribute to increased CA activity in patients with OSA. Along these lines it is worth mentioning that CA enzyme inhibition has been shown to reduce StHCO_3_^-^ concentration in patients with sleep disordered breathing [9, 22, 23] and that we previously have demonstrated an association between CA activity and the severity of OSA [3].

A particularly interesting finding in the current study was the strong association between StHCO_3_^-^ and hypertension status or diastolic blood pressure. It may be argued that this association could be explained by the well-established link between OSA and hypertension [5]. However, our data suggest that StHCO_3_^-^ was linked to hypertension independently of the AHI. Only few studies have addressed the possible association between StHCO_3_^-^ and hypertension. In a population based study of middle-aged non-obese females, lower plasma StHCO_3_^-^ was associated with an elevated incidence of hypertension [24]. A small experimental study of oral sodium bicarbonate induced approximately 5 mmHg reduction of systolic blood pressure [25] whereas other studies did not [26, 27]. However, these studies did not address subjects with the acute blood gas changes that characterize the OSA condition. In fact, our data suggest that the association between hypertension and StHCO3^-^ is mainly confined to subjects with severe OSA. As previously stated several different mechanisms, including increased renal re-absorption of StHCO_3_^-^, extended CO_2_ loading and/or increased CA activity, could all have increased of StHCO_3_^-^ in OSA [3, 18, 28]. In fact, a positive association between whole blood CA activity and blood pressure has been reported [3]. In addition, CA inhibition by zonisamide in OSA patients reduced both the AHI and the systolic blood pressure [9]. The CA inhibitor acetazolamide and hydrochlorothiazide induced vasodilation by an activation of calcium activated potassium channels [7, 29] or via a modulation of nitric oxide metabolism activity [30]. It cannot be excluded that the increase of StHCO3^-^ in our study might have resulted from the effect of hypertension on renal StHCO3^-^ reabsorption. However, this is less likely the explanation considering that StHCO3^-^ was elevated only in the severe OSA group with hypertension. We therefore propose that increased CA activity in OSA may provide a novel intermediary mechanism for hypertension development in OSA.

Our study has both strengths and limitations. First, this large predominently male clinical OSA cohort applied a rigorous and unique control of important confounders of StHCO_3_^-^ like arterial blood gas samples and pulmonary function tests. Second, blood pressure and hypertension status were carefully assessed during daytime as part of the study protocol. Sleep disordered breathing was assessed with a contemporary polygraphy recording device on two consecutive nights in order to exclude inaccuracy of the AHI value due to a first night effect [31]. Weaknesses include a lack of quantitative data on overnight hypoxic events like oxygen desaturation in the multivariate analyses. However, nocturnal hypoxic exposure was captured as 4% oxygen desaturation events that were used for compution of AHI. Another weakness in this study is the lack of detailed information on the type of antihypertensive medication. It cannot be excluded that prescribed antihypertensive medication, e.g. diuretics such as hydrochlorothiazide, might have influenced the association between StHCO_3_^-^ and blood pressure although the influence of thiazides on StHCO_3_^-^ is likely to be very limited [32]. Information on CA was not available in this retrospective study. Finally, the cross sectional design of our study does not allow conclusions about the causality of the demonstrated associations.

## Conclusions

It is concluded that StHCO_3_^-^ concentration was independently associated with AHI, a hypertension diagnosis and office diastolic blood pressure in OSA patients. The potential causal relationship (s) behind these associations remain unclear. Higher StHCO_3_^-^ was linked to more intense OSA indicating that high or upregulated CA activity is associated with OSA. In addition, StHCO_3_^-^ was associated with hypertension independent of sleep apnea suggesting a novel CA–related mechanism for blood pressure regulation in hypertensive OSA patients.

## Additional file

Additional file 1: Table S1. Normotensive patient characteristics across StHCO3^-^ quartiles (*n* = 388). **Table S2.** Association between LogStHCO_3_^-^ and systolic blood pressure in a generalized linear model. **Table S3.** Association between LogStHCO_3_^-^ and diastolic blood pressure in a generalized linear model. **Figure S1.** Relationship between pCO_2_ and stHCO_3_^-^ (Spearman correlation). **Figure S2.** Relationship between apnea hypopnea index and stHCO_3_^-^ (Spearman correlation). (DOCX 107 kb)

## Abbreviations

AHI: Apnea hypopnea index
ANOVA: One-way analysis of variance
BMI: Body mass index
CA: Carbonic anhydrase
CO_2_: Carbon dioxide
DBP: Diastolic blood pressure
OHS: Obesity-hypoventilation syndrome
OSA: Obstructive sleep apnea
SBP: Systolic blood pressure
StHCO3^-^: Bicarbonate
