# Supplementary material for: Independent associations between arterial bicarbonate, apnea severity and hypertension in obstructive sleep apnea
Source: Respir Res. 2017 Jun 28;18:130. doi: 10.1186/s12931-017-0607-9 (PMC5490198; doi:10.1186/s12931-017-0607-9)

**Independent associations between arterial bicarbonate, apnea severity and hypertension in obstructive sleep apnea**

Davoud Eskandari^1^, Ding Zou^1^, Ludger Grote^1^, Hartmut Schneider^2^, Thomas Penzel^3^, and Jan Hedner^1^

^1^Center for Sleep and Vigilance Disorders, Department of Internal Medicine and Clinical Nutrition, Sahlgrenska Academy, University of Gothenburg, Gothenburg, Sweden;

^2^Johns Hopkins Sleep Disorders Center, Division of Pulmonary and Critical Care Medicine, Johns Hopkins University, Baltimore, Maryland, USA;

^3^Interdisciplinary Center of Sleep Medicine, Charité-Universitätsmedizin Berlin, Berlin, Germany.

Additional file:

Table S 1: Normotensive patient characteristics across StHCO3^-^ quartiles (n=388)

|  | Q1  [17.5-22.9] mmol/l | Q2  [23.0-24.2] mmol/l | Q3  [24.3-25.4] mmol/l | Q4  [25.5-29.7] mmol/l | P value  (ANOVA) |
| --- | --- | --- | --- | --- | --- |
| Male sex (%) | 90 | 89 | 95 | 95 | n.s.* |
| Age (yrs) | 50 (11) | 51 (10) | 47 (10) | 50 (10) | 0.055 |
| Body mass index (kg/m^2^) | 29 (6) | 28 (5) | 28 (4) | 29 (5) | n.s. |
| Smoking (%) | 29 | 28 | 39 | 34 | n.s.* |
| Systolic BP (mmHg) | 136 (16) | 133 (13) | 135 (14) | 139 (17) | 0.051 |
| Diastolic BP (mmHg) | 87 (9) | 86 (10) | 88 (9) | 88 (11) | n.s. |
| Heart rate (bpm) | 73 (11) | 71 (10) | 72 (11) | 73 (10) | n.s. |
| Apnea hypopnea index (n/h) | 26 (21) | 25 (21) | 24 (21) | 31 (22) | 0.094 |
| FEV_1_/FVC (%) | 82 (5) | 82 (6) | 83 (6) | 82 (6) | n.s. |
| pH | 7.41 (0.03) | 7.41 (0.02) | 7.42 (0.02) | 7.42 (0.02) | <0.001 |
| pO_2_ (kPa) | 11.0 (1.2) | 10.8 (1.0) | 10.7 (1.0) | 10.4 (1.1) | 0.001 |
| pCO_2_ (kPa) | 4.62 (0.30) | 4.97 (0.26) | 5.20 (0.24) | 5.44 (0.22) | <0.001 |
| StHCO_3_^-^ (mmol/l) | 21.7 (1.2) | 23.7 (0.4) | 24.9 (0.3) | 26.4 (0.8) | - |

*Fisher’s exact test; BP= blood pressure; bpm= beat per minute; FEV1/FVC= forced expiratory volume at 1 second interval / forced vital capacity; pO_2_= arterial partial pressure of oxygen; pCO_2_= arterial partial pressure of carbon dioxide; StHCO_3_^-^ = arterial standard bicarbonate.

Table S2. Association between LogStHCO_3_^-^ and systolic blood pressure in a generalized linear model.

|  | Beta Value | Standard Error | 95% confidence interval | P-value |
| --- | --- | --- | --- | --- |
| Male sex | -1.11 | 2.72 | -6.45 – 4.22 | n.s. |
| Age (years) | 0.46 | 0.08 | 0.32 – 0.61 | <0.001 |
| Body mass index (kg/m^2^) | 1.28 | 0.14 | 1.00 – 1.56 | <0.001 |
| Smoking | -2.66 | 1.52 | -5.65 – 0.33 | 0.081 |
| Alcohol | 2.35 | 1.42 | -0.44 – 5.13 | n.s. |
| Moderate vs. mild OSA | 3.16 | 1.76 | -0.29 – 6.60 | 0.072 |
| Severe vs. mild OSA | 5.91 | 1.66 | 2.65 – 9.18 | <0.001 |
| PO_2_ (kPa) | 0.13 | 0.09 | -0.05 – 0.32 | n.s. |
| PCO_2_ (kPa) | 0.26 | 0.31 | -0.34 – 0.86 | n.s. |
| LogStHCO_3_^-^ | 17.07 | 22.34 | -26.72 – 60.86 | n.s. |

Table S3. Association between LogStHCO_3_^-^ and diastolic blood pressure in a generalized linear model.

|  | Beta Value | Standard Error | 95% confidence interval | P-value |
| --- | --- | --- | --- | --- |
| Male sex | 3.46 | 1.70 | 0.12 – 6.79 | 0.042 |
| Age (years) | 0.08 | 0.05 | -0.01 – 0.17 | 0.085 |
| Body mass index (kg/m^2^) | 0.71 | 0.09 | 0.54 – 0.89 | <0.001 |
| Smoking | -1.95 | 0.95 | -3.82 – -0.08 | 0.041 |
| Alcohol | 0.43 | 0.89 | -1.30 – 2.17 | n.s. |
| Moderate vs. mild OSA | 1.75 | 1.10 | -0.40 – 3.91 | n.s. |
| Severe vs. mild OSA | 2.29 | 1.04 | 0.25 – 4.33 | 0.028 |
| PO_2_ (kPa) | 0.03 | 0.06 | -0.08 – 0.15 | n.s. |
| PCO_2_ (kPa) | 0.04 | 0.19 | -0.34 – 0.41 | n.s. |
| LogStHCO_3_^-^ | 27.60 | 13.96 | 0.24 – 54.96 | 0.048 |

Figure S1. Relationship between PCO_2_ and stHCO_3_^-^ (Spearman correlation).


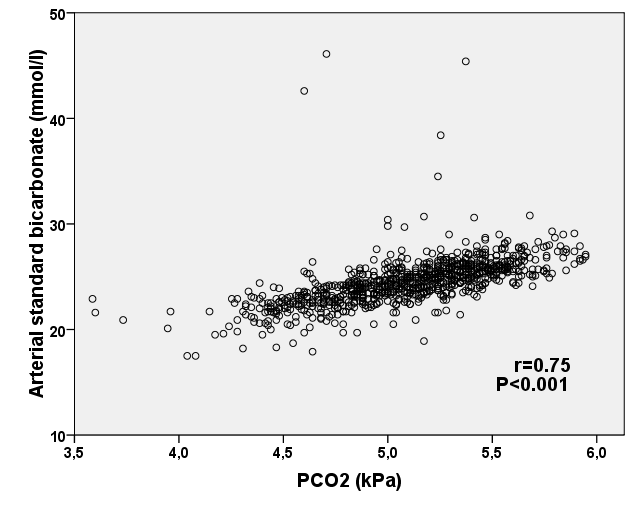


Figure S2. Relationship between apnea hypopnea index and stHCO_3_^-^ (Spearman correlation).


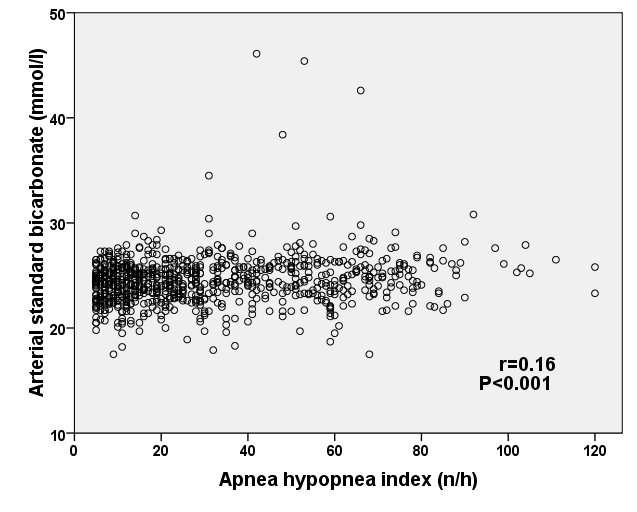

Supplement: Additional file 1: Table S1. — Normotensive patient characteristics across StHCO3- quartiles (n = 388). Table S2. Association between LogStHCO3 - and systolic blood pressure in a generalized linear model. Table S3. Association between LogStHCO3 - and diastolic blood pressure in a generalized linear model. Figure S1. Relationship between pCO2 and stHCO3 - (Spearman correlation). Figure S2. Relationship between apnea hypopnea index and stHCO3 - (Spearman correlation). (DOCX 107 kb) [file 12931_2017_607_MOESM1_ESM.docx]
